# *Geranium sylvaticum* increases pollination probability by sexually dimorphic flowers

**DOI:** 10.1002/ece3.9670

**Published:** 2022-12-28

**Authors:** Jaakko O. S. Soininen, Minna‐Maarit Kytöviita

**Affiliations:** ^1^ Department of Biological and Environmental Sciences, Faculty of Mathematics and Science University of Jyväskylä Jyväskylä Finland

**Keywords:** disruptive selection, flower size, *Geranium sylvaticum*, gynodioecy, pollination, sexual dimorphism

## Abstract

Sexual dimorphism is expressed as different morphologies between the sexes of a species. Dimorphism is pronounced in gynodioecious populations which consist of female and hermaphrodite individuals. The small size of female flowers in gynodioecious species is often explained by resource re‐allocation to seed production instead of large flowers. However, pollinator attraction is critical to female fitness, and factors other than resource savings are needed to explain the small size of female flowers. We hypothesized that the floral size dimorphism in the perennial gynodioecious *Geranium sylvaticum* (L.) is adaptive in terms of pollination. To test this “pollination hypothesis,” we video recorded the small female and large hermaphrodite *G. sylvaticum* flowers. We parameterized floral visitor behavior when visiting a flower and calculated pollination probabilities by a floral visitor as the probability of touching anther and stigma with the same body part. Pollination probability differed in terms of flower sex and pollinator species. Bumblebees had the highest pollination probability. The small female flowers were more likely to receive pollen via several pollinator groups than the large hermaphrodite flowers. The pollen display of hermaphrodites matched poorly with the stigma display of hermaphrodites, but well with that of females. Although the small size of female flowers is commonly explained by resource re‐allocation, we show that sexual dimorphism in flower size may increase the main reproductive functions of the females and hermaphrodites. Dimorphism increases pollination probability in females and fathering probability of the hermaphrodites likely driving *G. sylvaticum* populations towards dioecy.

## Graphical abstract

In this work, we add to the understanding of the function of sexual dimorphism in the plant *Geranium sylvaticum*. Our results demonstrate that the sexually different flower size in the female and hermaphrodite flowers are optimized for inter‐sexual pollen transportation via the pollinator insects.

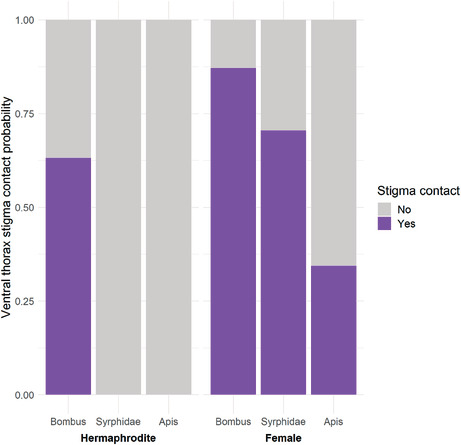

## 1 INTRODUCTION

Sexual dimorphism in plants refers to the morphological differences between sexes. Sexual differences in vegetative traits are usually absent. Although some intrinsic differences are present in the primary sex organs, sexual dimorphism refers to, and is most pronounced in, differences in flower or inflorescence morphology (Ashman, 2005). Sexual dimorphism is most notable in dioecious and gynodioecious species. In dioecious species, the two sexes are expressed in different plant individuals. Gynodioecious populations consist of female individuals that bear flowers with only the female function, and hermaphrodite individuals with both the female and male function (Ågren & Willson, 1991; Eckhart & Chapin, 1997; Miller & Venable, 2003). Approximately 6% of angiosperms are dioecious (Renner & Ricklefs, 1995) and gynodioecy is present in 2.2% of angiosperm families, while 0.5% of dicot species are gynodioecious (Godin & Demyanova, 2013). Gynodioecious species are proposed to arise as cytoplasmic determinants followed by mutations that cause the loss of the male function in hermaphrodite flowers (Budar et al., 2003; Schnable & Wise, 1998).

Despite the understanding of the mechanisms of how gynodioecy may arise, it is challenging to explain for many reasons. The loss of male function entails that females lose half of the reproductive fitness of the hermaphrodites associated with pollen. Due to this inborn disadvantage of the females, females must compensate for the lost half of their reproductive fitness in comparison to hermaphrodites (Lewis, 1941; Lloyd, 1976). In the absence of alleviating factors, the increased contribution to the gene pool of the offspring should at least account for the fitness derived from pollen. The female compensation in fertility is usually less than the required compensation which in some cases can be expected as high as 200%, although the compensation depends on the sex ratio of the population and the mechanism of male sterility (Charlesworth & Charlesworth, 1978; Lewis, 1941). Increased seed viability and increased offspring fitness resulting from avoidance of inbreeding depression in females have been suggested to reduce female disadvantage further (Dufay & Billard, 2012; Puterbaugh et al., 1997). Females may gain benefits by avoiding inbreeding depression (Baker, 1959; Charlesworth & Charlesworth, 1978; Sakai et al., 1997), but the resulting benefit is difficult to evaluate. As some gynodioecious species show little inbreeding depression (Mutikainen & Delph, 1998), female advantage by cross‐pollination may not be universal.

The consequent loss of fitness along the male function is not the only problem posed by females in gynodioecious species. Fitness in females is critically dependent on pollinators visiting female flowers after visiting the pollen‐bearing hermaphrodite flowers. As a general rule, in sexually dimorphic species, the female flowers are significantly smaller than those of the larger, showier hermaphrodite flowers (Ågren & Willson, 1991; Barret & Hough, 2013; Miller & Venable, 2003). Female flowers may also provide less nectar to the pollinators (Delph & Lively, 1992; Klinkhamer et al., 1991; Varga, Nuortila, & Kytöviita, 2013) and intrinsically lack pollen. Because pollinators strongly discriminate between flowers and prefer large and showy (Bond & Maze, 1999; Martin, 2004), symmetric flowers (Moller, 1995) with ample rewards (Delph & Lively, 1992; Varga & Kytöviita, 2010), hermaphrodite flowers are predicted to be selected for these traits in promotion of their male function (Vaughton & Ramsey, 1998). In line with the showiness and rewards, insects visit hermaphrodite flowers more frequently than those of the females in most gynodioecious species (Asikainen & Mutikainen, 2005a; Cuevas et al., 2008; Van Etten & Chang, 2014; Varga & Kytöviita, 2010).

Furthermore, many pollinators exhibit flower constancy, i.e., behavior where the pollinator learns fidelity toward a specific rewarding plant species or morph (Waser, 1986). Flower constancy is proposed to be based on the handling skills required to access rewards (Ishii & Kadoya, 2016), visual appearance (Gegear & Laverty, 2005; Ishii & Masuda, 2014), and olfactory cues (Laska et al., 1999; Wright & Schiestl, 2009) of the flower that the pollinator learns to favor. Flower constancy is considered an important aspect of the evolutionary ecology of plant–pollinator interactions as it improves the pollination services received by the plant. For instance, it reduces the probability of clogging the stigma with the pollen of other species (Morales & Traveset, 2008; Muchhala & Thomson, 2012). On the other hand, it reduces the amount of wasted pollen in terms of transport to intraspecific recipient flowers (Schmid et al., 2016). The flower constancy and consequent potential passing over the females by the pollen carriers are aggravated by the fact that there are usually fewer females in a gynodioecious population (Asikainen & Mutikainen, 2003; Chang, 2006). This often leads to minority disadvantage (Levin, 1972) and females receive less visits by pollinators which mainly forage the most common morphs (Levin, 1972; Van Etten & Chang, 2014). Females cannot equal hermaphrodites in frequency (Charlesworth & Charlesworth, 1978) to escape minority disadvantage (Levin, 1972), but females could attract pollinators more efficiently (Glaettli & Barrett, 2008) and counteract the minority disadvantage by increased floral attraction. Furthermore, female flowers may compensate for smaller flower size by remaining in the receptive phase longer (Ashman & Stanton, 1991). Despite these potential counteractive measures, females have been frequently shown to receive fewer pollinator visits than hermaphrodites or males (Asikainen & Mutikainen, 2005a; Bond & Maze, 1999; Cuevas et al., 2008; Van Etten & Chang, 2014; Varga & Kytöviita, 2010) although not universally in all studies (e.g., Cervantes et al., 2018).

In hermaphrodite flowers, the male function may pose different evolutionary selection pressures on floral morphology than the female function (Barret, 2002). Hermaphrodites are subject to the cost of increased inbreeding depression resulting from self‐pollination (Charlesworth & Charlesworth, 1987; Varga, Vega‐Frutis, & Kytöviita, 2013). Arising from the different evolutionarily stable strategies in the sexes, pollinator‐limited males are also proposed to allocate on floral display and reward (Thomson & Brunet, 1990). In gynodioecious populations, hermaphrodites gain most of their fitness through the male function due to the presence of females (Charlesworth, 1981; Lloyd, 1976;Vamosi & Otto, 2002). This should select for larger floral displays and pollen production in hermaphrodites (Vaughton & Ramsey, 1998) because the male function is promoted by pollen export and thus ultimately attractiveness to pollinators.

Most studies explain sexual flower size dimorphism in gynodioecious species by different aspects of resource allocation and trade‐offs (e.g., Ashman, 1992, 1994; Delph et al., 1996; Miller & Venable, 2003). Seed production demands a substantial portion of plant resources (Ashman, 1992). For example, Ashman (1992) found that *Sidalcea oregana* plants allowed to make seeds allocated 20% less biomass to floral structures, and in turn, plants that were not, produced 40% more floral biomass the next year than the plants that were allowed to produce seeds the first year. The higher allocation in seed set in females vs. hermaphrodites has been suggested to be possible via enhanced resource allocation to female function (Ashman, 1994; Chang, 2006). The decreased size of the corolla as well as the loss of stamens in females may leave more resources for seed production (Ashman, 1994; Eckhart, 1992). We argue that the benefit gained from re‐allocating floral biomass to seed mass is inadequate given that the small flower size handicaps pollination (Bond & Maze, 1999; Martin, 2004). It would be more economic for the plant to re‐allocate resources to seeds from less critical sources such as older parts of foliage or roots rather than the critical floral display. The difference in flower size between sexes is a general phenomenon, and we propose that factors other than resource savings are needed to explain the apparent mismatch between costs and benefits of the smaller flower size in females in gynodioecious plant populations.

In this work, we explore an alternative, but not necessarily exclusive hypothesis to explain sexual dimorphism. We focus on *Geranium sylvaticum*, a gynodioecious perennial plant with sexually dimorphic populations consisting of female and hermaphrodite individuals. The female flowers are smaller than the hermaphrodite ones (Asikainen & Mutikainen, 2005a; Varga & Kytöviita, 2010), provide less nectar (Varga, Nuortila, & Kytöviita, 2013), and naturally no pollen as a reward for pollinators. The female flowers are visited less frequently by insect visitors (Asikainen & Mutikainen, 2005a; Varga & Kytöviita, 2010). We hypothesize that the small size of female flowers in *G. sylvaticum* is adaptive because it increases pollination probability in females and thus the fitness gained by female function in females and male function in hermaphrodites. We test this “pollination hypothesis” by comparing the probability of pollen transport from anther to receptive stigma (I) between hermaphrodite flowers and (II) between hermaphrodite and female flowers. Support for the hypothesis that sexual dimorphism is adaptive will be evidenced if (I) is smaller than (II). Furthermore, we compare the probability of pollen transport from an anther to a stigma by the most common floral visitors of *G. sylvaticum*. We hypothesize that the small size of female flowers in *G. sylvaticum* is an adaptation to pollination by bumblebees and expect that bumblebees rather than the other common floral visitors are responsible for pollen transport between flowers. Each insect visitor has characteristics that determine its specific pollination efficiency (Motten, 1986). These are how frequently and how faithfully the insect visits a given host, how much pollen it carries during visits, and how the visitor morphology and foraging behavior match with the flower morphology. In the present work, we investigate the latter point related to visitor behavior and how it matches the morphology of the two sexes of *G. sylvaticum*.

## 2 MATERIALS AND METHODS

### 2.1 Study organism

*Geranium sylvaticum* (L.) is a self‐compatible perennial with Eurasian distribution (Stroh, 2014). *Geranium sylvaticum* is common in meadows but thrives also in shade (Korhonen et al., 2004), in particular when nutrient availability is high (Hokkanen, 2003). The plant is gynodioecious, and the proportion of female plants varies between 0% and 23% between populations (Asikainen & Mutikainen, 2005a; M.‐M. Kytöviita, personal observations). Both female and hermaphrodite flowers offer nectar as a reward for pollinators (Varga, Nuortila, & Kytöviita, 2013). The fruit matures in 3 weeks after fertilization and is a schizocarp with five locules and the maximum number of seeds per fruit is five.

### 2.2 Field measurements

We estimated pollen transport probabilities by quantifying floral visitors and their behavior in detail in video recorded *G. sylvaticum* plants. The plants were growing in an experimental site of the University of Jyväskylä established in an old field year 2008 at Konnevesi Finland (62°35′17.4″N 26°14′03.2″E). Altogether seven female plants and 13 hermaphrodite plants were video recorded when in full bloom between June 14 and 18, 2021. The plants were of the same age and size and were composed of 34 floral shoots on average. Alternating between random female and hermaphrodite plants, a portion of the inflorescences were recorded on average in 40‐min intervals. Multiple cameras ensured that the temporal variability in insect activity did not affect behavior in the sexes differently.

**FIGURE 1:**
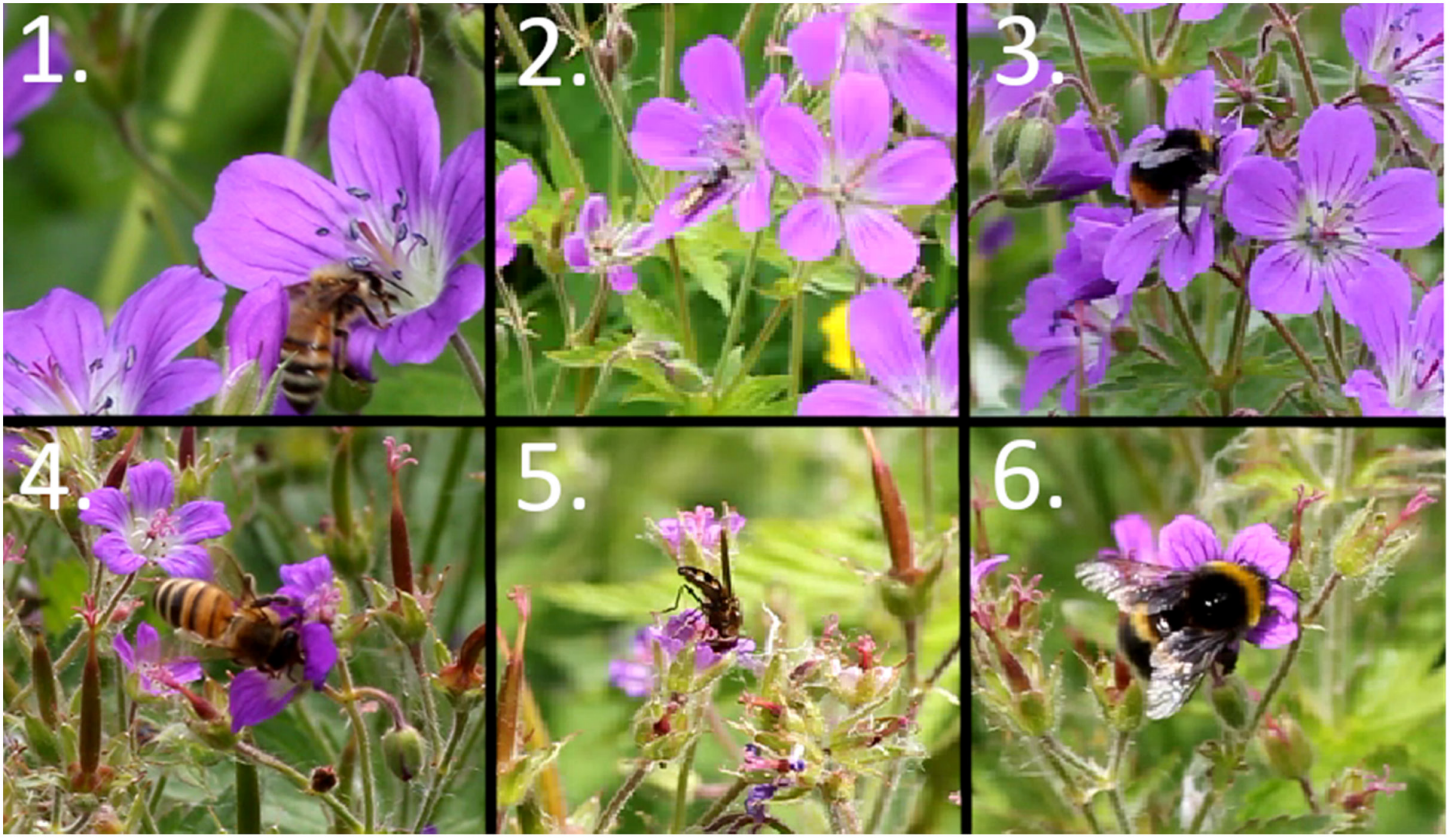
Examples of the video material illustrating the behavior of different insect pollinators and the different sexes of the plant *Geranium sylvaticum*. 1. *Apis mellifera* visiting a non‐receptive hermaphrodite flower touching the anthers with the head. 2. A Syrphidae resting in a receptive hermaphrodite flower. The fly slips under the anthers and makes little contact with reproductive structures. 3. *Bombus pratorum* visiting a receptive hermaphrodite flower. The bee has climbed over the reproductive structures so that the thorax contacts both the anthers and the stigma. 4. *Apis mellifera* visiting a female flower. Due to the small size of the flower, the bee reaches the nectaries over the receptive stigma and touches the stigma surfaces with ventral side of the thorax. 5. A Syrphidae visiting a female flower making contact with the stigma while reaching the nectaries across the stigma. 6. *Bombus soroeensis* on a female flower. Due to the small size of the flower, the bee has planted itself over the flower for easiest access to nectaries. It touches the stigma with its ventral side of thorax.

The plants were video recorded during the most active period of insects (9:00 a.m. to 6:00 p.m.). The image was focused so that 10–15 fully open flowers in a plant could be followed simultaneously with a sufficient accuracy to distinguish the pollinator behavior (Figure 1). The hardware used for recording included Canon EOS 550D digital camera with 55–250 mm objective set to 250 mm, as well as portable computer‐run cameras with the use of the application OBS studio ver. 26.1.1. (64 bit). Altogether 30 h of video data were gathered on the 20 plant individuals, of which a total of 13 h were gathered on the hermaphrodite plants, and 17 h on the female plants. Female plants were recorded more to compensate for the expected lesser visitation rates in females versus hermaphrodites.

### 2.3 Visitation parametrization

The flower‐visiting insects were assigned to seven groups (hereafter visitor groups) which consisted of bumblebees in the genus *Bombus* (hereafter *Bombus*), honeybees *Apis mellifera* (L.) (hereafter *Apis*), hoverflies of the family Syrphidae (hereafter Syrphidae), and solitary Hymenoptera, Diptera, Hemiptera, and Coleoptera. Syrphidae were intentionally separated from Diptera in general due to their distinct behavior and abundance, and *Apis mellifera* from other eusocial bees because *Apis mellifera* is farmed in Finland and does not occur naturally.

The behavior of an insect was parameterized so that a contact with the reproductive surface of an anther or stigma in the flower was noted along with the body part of the insect that had made the contact. The body parts were classified as follows: head ventral, head dorsal, foot, leg, thorax ventral, thorax dorsal, abdomen ventral, and abdomen dorsal. We only report visitations where it was possible to distinguish the movements of an insect within a flower and whether it had contacted the floral reproductive organs. In addition to the movements of the insect, the stigma phase (receptive/non‐receptive) and the time of visit were recorded. Visitation frequencies by insect group per hour were extrapolated by following the flowers visible on the screen for the length of the video. These data are not based on the sexual organ contact data as all visitations were usable to estimate the data on frequency.

When the fruits were ripe in August, the schizocarps were collected and dried (60°C, 12 h). Based on the seed scars in the schizocarps, the average seed production per flower, total seed production per plant, and the ratio of undeveloped‐to‐developed schizocarps were counted.

### 2.4 Data analysis

In the probability estimations and statistical analyses, we only used the contacts with the ventral side of the insect's body (i.e., ventral side of head, thorax, and abdomen). This is because *G. sylvaticum* flowers are sternotribic (Kozuharova, 2002), and dorsal contacts by the insects were ineligible. Dorsal contacts would not transmit pollen; although an insect could touch anthers with dorsal side, it could not land upside down on the stigma. Correspondingly, there were a few dorsal anther contacts (mainly with the head), but no dorsal stigma contacts in the video material.

The probability of pollination was calculated and defined as the probability of an insect contacting an anther with a certain part of the body and contacting the stigma of a flower with the same body part.

The probabilities of pollen transport between the compared groups were calculated with the basic formula of the probability of two independent events:

$$P\left(A\cap B\right)=P\left(A\right)\times P\left(B\right)$$

where P(A) is the probability of anther contacts (anther contacts/visits) in the visitor group, and P(B) is the probability of contacting stigma in the receptive phase (stigma contacts/visits) in the same visitor group. This method of calculating pollination probability focuses on insect behavior when visiting a flower but does not estimate the holistic pollinator efficiency (Motten, 1986).

The probabilities of pollination were calculated separately in the two plant sexes (females and hermaphrodites) and the different pollinator groups, so that ventral contacts to anthers and stigma were taken into account, respectively, for all body parts (head, thorax, and abdomen) and summed to get the final pollination likelihood.

The data were statistically analyzed as follows. The data on anther or stigma contacts were analyzed with generalized linear regression model (GLM, with the logit link function and binomial family distribution). In the GLM, the frequency of ventral stigma or anther contact (yes/no) was set as the response variable and the plant sex (not in the model for anther contact) and visitor group as predictor variables. The visitor group analysis was repeated by setting each visitor group as the reference level to compare the visitor groups with each other. In the analyses, visits by Diptera, Coleoptera, Hemiptera, and solitary Hymenoptera were not tested as there were not enough visits to draw reliable conclusions. For visitations per flower per hour frequency data, negative binomial generalized linear model with log link function was used, with visitation rate per flower per hour set as the response variable and sex and the number of floral shoots were used as predictor terms. Analyses for effects on mean or overall seed production were conducted with GLM using the Gaussian family distribution with logit link function for the response variable, which was either the mean seed production per flower in a plant or total seed production. Models with the ratio of undeveloped to developed schizocarps as the response variable were conducted with quasibinomial distribution family and logit link function. As predictor terms, visitation rates by insect groups per flower in an hour, sex, and number of floral shoots were used, depending on the optimal model determined by AIC values and/or the distribution of residuals.

Within each insect group, the differences in frequencies in stigma contacts between plant sexes were analyzed with two‐sample *Z*‐test for probabilities using a subset of data at a time containing only one visitor group. To test the statistical significance of the differences in the pollination probabilities between the plant sexes, we resampled the data by randomly selecting pairs of anther contact (0/1) and stigma contact (0/1) iterated 5000 times. If there was a contact (1) on both anther and stigma, it was taken as a probable pollination event. These resampled data conformed to the aforementioned grouping so that all combinations of sex, body part, and visitor class were present. The random‐pair data were used in two‐sample *Z*‐test to analyze the differences in the anther–stigma random frequencies of successful pollinations between the female and hermaphrodite groups in the respective body parts (head ventral, thorax ventral, and abdomen ventral) and in the respective visitor groups (Syrphidae, *Bombus* and *Apis*).

The data were analyzed using the statistical programming software R, ver. 4.0.2. (64 bit).

## 3 RESULTS

Altogether, we recorded 536 insect visits in the study plants. Of the visits, 406 were observed in flowers with receptive stigma. In *G. sylvaticum* flowers, the stigma lobes are closed when non‐receptive and open in the receptive female phase (Varga, Nuortila, & Kytöviita, 2013). The recorded insects belonged to a range of taxa: bumblebees (*Bombus pratorum* (L.), *B. soroeensis* (Fabricius), *B. lucorum coll*. (L.), *B. sporadicus* (Nylander), *B. pascuorum* (Scopoli), *B. hypnorum* (L.), *B. terrestris* (L.), *B. bohemicus* (Seidl), and *B. lapidarius* (L.)), honey bee (*Apis mellifera* (L.)), syrphid flies (e.g., *Sphaerophoria scripta* (L.), *Syrphus ribesii* (L.), *Microdon* sp., *Cheilosia* sp., and *Helophilus pendulus* (L.)), and solitary Hymenoptera (*Lasioglossum* sp. and *Corynis obscura* (F.)) accompanied with various species of Diptera; various Brachycera and some small Nematocera. At the site, occasionally Coleoptera such as *Zacladus geranii* (Paykull), *Corizus hyoscyami* (L.), *Coreus marginatus* (L.), *Pentatoma rufipes* (L.), and *Dolycoris baccarum* (L.) were observed as well as some butterflies (*Vanessa atalanta* (L.), *Anthocharis cardamines* (L.), *Aglais io* (L.), and *Araschnia levana* (L.)) and some moths (Geometridae). In terms of the plant sexes (hermaphrodite/female), we recorded 68/163 visits by *Bombus*, 63/88 by Syrphidae, 9/1 by Solitary Hymenoptera, 45/32 by *Apis*, and 14/15 by Diptera, respectively. It should be noted that these data are simply the recorded visitations that were distinguished in the videos, and do not represent differences in frequency of visitations between sexes, but the number of visits we video recorded (size of data) and on which the contact probability calculations are based on.

### 3.1 Visitation frequency

Pooling all of the insect groups, the female plants received 12.5 visits per flower per hour and hermaphrodites received 20.14 visits per hour. However, these overall visitation rates were not statistically significantly different between the sexes (df = 45, AIC = 354.58, Estimate = 0.48, *z* = 1.26, *p* = .208). Visitation frequencies by the three focal insect groups in females/hermaphrodites were 5.44/9.33 in *Bombus* (df = 45, AIC = 981.11, Estimate = 0.53, *z* = 4.88, *p* < .001), 1.48/2.61 in *Apis* (df = 45, AIC = 351.13, Estimate = 0.56, *z* = 2.69, *p* = .007), and 3.8/4.08 in Syrphidae (df = 45, AIC = 218.43, Estimate = 0.07, *z* = 0.126, *p* = .899). Visitation rates by *Bombus* and *Apis*, but not by Syrphidae, were statistically significantly smaller in females than in hermaphrodites.

### 3.2 Anther contacts

Overall, the anthers in hermaphrodite flowers had .34 probability to be contacted during a visit. The probability to contact anthers with the ventral side of the body of an insect during a floral visit was .78 in *Bombus*, .07 in *Apis*, and .06 in Syrphidae.

The probability to contact anthers with ventral side by members of Syrphidae did not differ from that of *Apis* (df = 69, AIC = 52.1, Estimate = 0.48, *z* = 0.38, *p* = .70) but was lower than that of *Bombus* (df = 69, AIC = 52.1, Estimate = 4.36, *z* = 3.74, *p* < .01). The probability to contact anthers with ventral side by *Apis* was inferior to that by *Bombus* (df = 69, AIC = 52.1, Estimate 3.88, *z* = 4.20, *p* < .01). Ventral anther contact hierarchy was thus established as *Bombus* > Syrphidae, *Apis*.

**FIGURE 2:**
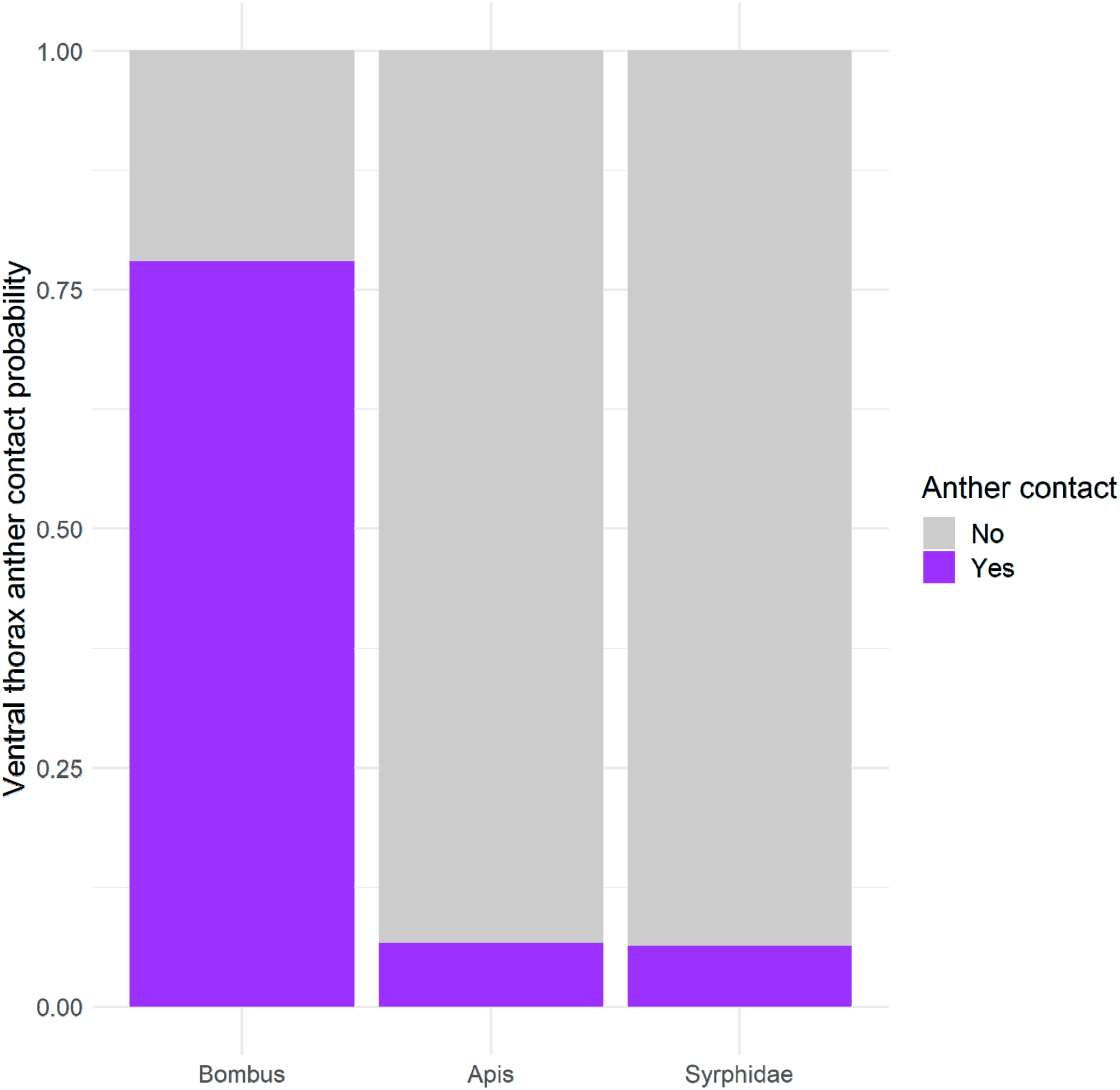
Average anther contact probabilities by different visitor groups (*Bombus, Apis*, and Syrphidae) in the hermaphrodite *Geranium sylvaticum* flowers (*N* = 199). Anther contacts are binary (yes, no), the contacts represent ventral thorax contacts only.

The visitor probabilities to contact anthers are visualized in Figure 2.

### 3.3 Stigma contacts

The stigma contacts were influenced by plant sex. Females had an overall higher probability of receiving a contact to the receptive stigma by a floral visitor (*p* = .72) than hermaphrodites (*p* = .16) (df = 367, AIC = 331.75, Estimate = 2.50, *z* = 6.15, *p* < .01). The probability to contact a female/hermaphrodite receptive stigma with the ventral side in the main visitor groups was .87/.63 in *Bombus* (df = 180, AIC = 154.25, Estimate = 1.37, *z* = 2.60, *p* = .01), .34/<.001 in *Apis* (df = 58, AIC = 49.213, Estimate = 0.34, *z* = 3.76, *p* < .001), and .70/<.001 in Syrphidae (df = 108, AIC = 120.98, Estimate = 0.7, *z* = 7.18, *p* < .001).

Between visitor group comparisons revealed that in both sexes the stigma contact probability in the group Syrphidae was greater than in *Apis* (df = 367, AIC = 331.75, Estimate = −1.49, *z* = −3.59, *p* < .01) but less than in *Bombus* (df = 367, AIC = 331.75, Estimate = 2.94, *z* = −6.95, *p* < .01). Also, *Apis* had smaller probability to contact the stigma than *Bombus* (df = 367, AIC = 331.75, Estimate = 2.94, *z* = 6.95, *p* < .01). Stigma contact hierarchy was thus established as *Bombus >* Syrphidae > *Apis*.

**FIGURE 3:**
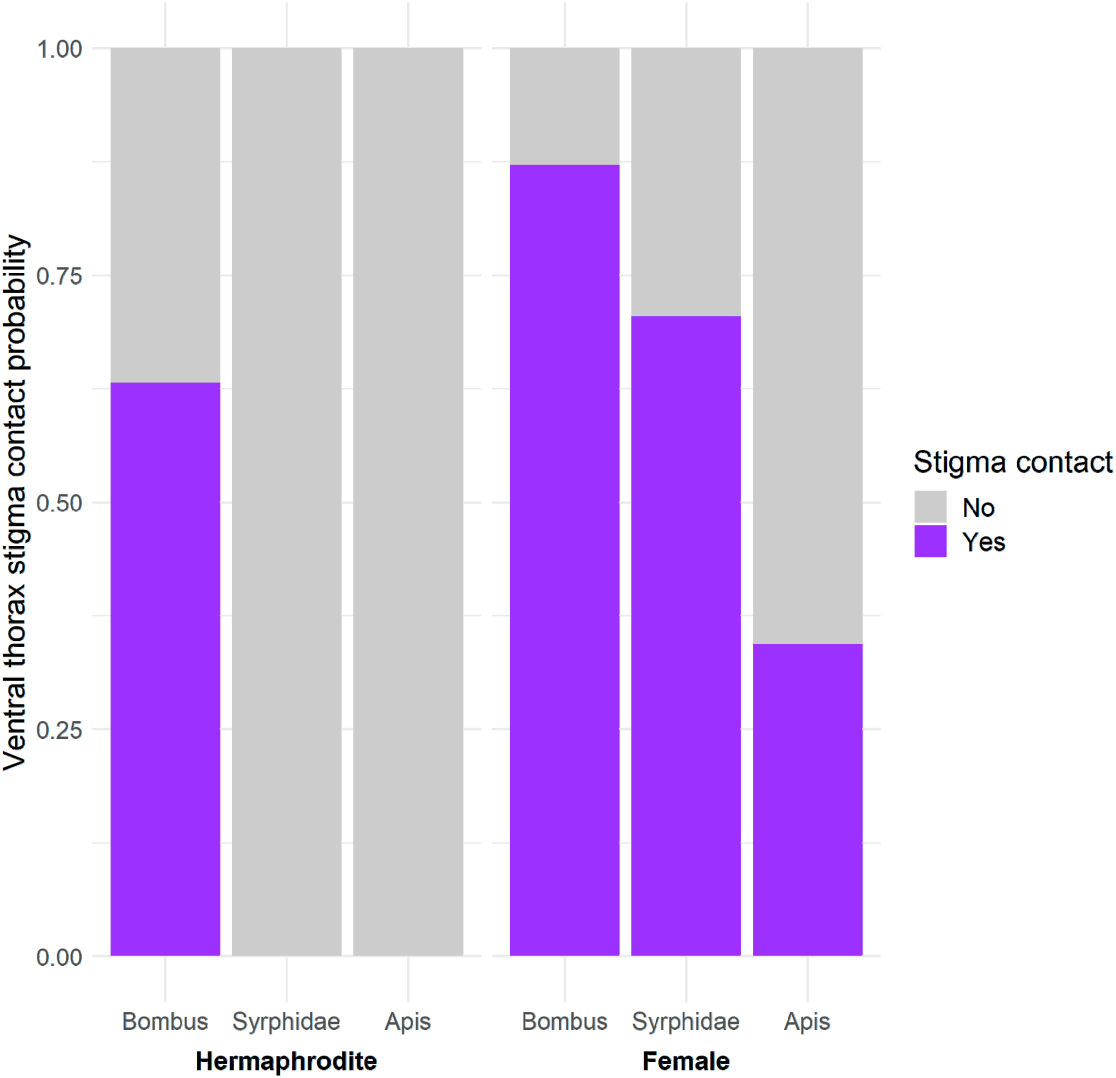
Average stigma contact probabilities by the different visitor groups (*Bombus, Apis*, and Syrphidae) in the hermaphrodite and female *Geranium sylvaticum* flowers. Stigma contacts are binary (yes, no), the contacts represent ventral thorax contacts only.

Visitor probabilities to make stigma contacts in female and hermaphrodite flowers are illustrated in Figure 3.

### 3.4 Pollination probability

The probability of transporting pollen from the anthers of a hermaphrodite plant to the stigma of hermaphrodite plant by a specific pollinator group during a single visit was .47 in *Bombus* and <.001 in the case of *Apis* and Syrphidae. The probability to make contact with the anthers of a hermaphrodite plant and then make a contact with the stigma in a female plant was .68 in terms of *Bombus*, .04 of Syrphidae, and .017 of *Apis*. The difference between the plant sexes was statistically significant in *Bombus* head ventral, thorax ventral, and abdomen ventral pollination probabilities (Table 1). Similarly, the pollination probability with *Apis* head ventral, thorax ventral, and abdomen ventral differed statistically significantly between the sexes (Table 1). In Syrphidae, pollination probabilities differed significantly between the sexes in head ventral and thorax ventral contacts (no data were recorded on abdomen ventral contacts; Table 1). In general, the hermaphrodite plants had inferior probability to be pollinated by any pollinator group compared to that of females. The statistical significances and test values between the different visitor groups and body parts in female/hermaphrodite plants are shown in Table 1.

**TABLE 1:** Statistics, probabilities, and *p*‐values from a two‐sample *Z*‐test for equality of proportions test depicting the comparisons between the probability to pollinate female (F) versus hermaphrodite (H) *Geranium sylvaticum* flowers in different visitor groups (rows) and their respective ventral side body parts (columns).

VISITOR CLASS	Head	Thorax	Abdomen
*Bombus*, F vs. H plants	*χ* ^2^ = 807.48, df = 1 *p*_(F)_ = .15, *p*_(H)_ < .001 *p* < .01	*χ* ^2^ = 252.96, df = 1 *p*_(F)_ = .64, *p*_(H)_ = .49 *p* < .01	*χ* ^2^ = 9.1, df = 1 *p*_(F)_ < .001 *p*_(H)_ = .002 *p* < .01
*Apis*, F vs. H plants	*χ* ^2^ = 2, df = 1 *p*_(F)_ < .001, *p*_(H)_ < .001 *p* = .13	*χ* ^2^ = 58.36, df = 1 *p*_(F)_ = .012, *p*_(H)_ < .001 *p* < .01	*χ* ^2^ = 13.08, df = 1 *p*_(F)_ = .003, *p*_(H)_ < .001 *p* < .01
Syrphid, F vs. H plants	*χ* ^2^ = 5.15, df = 1 *p*_(F)_ = .001, *p*_(H)_ < .001 *p* = .02	*χ* ^2^ = 168.84, df = 1 *p*_(F)_ = .34, *p*_(H)_ < .001 *p* < .01	NA

*Note*: Syrphidae abdomen ventral contact probabilities were not comparable as no contacts occurred within this group.

Without sex discrimination, the likelihood to contact anthers and then any stigma was .64 in *Bombus*, .04 in Syrphidae, and .01 in *Apis*.

The possibility for autogamous pollination occurred only in the visitor group *Bombus*. In 16.2% of visits, *Bombus* touched both the anthers and the stigma with the same body part, but taking the insect behavior within the flower into account it was estimated that only 5.4% of *Bombus* visits in the hermaphrodites could have potentially led to autogamous pollination.

According to the calculated probability values, the pollination probabilities in both plant sexes rank as *Bombus* > Syrphidae > *Apis* in the three main visitor groups.

### 3.5 Seed production

Hermaphrodites produced on average 1605 ± 996 seeds per plant and females 2654 ± 402. The difference between the sexes was statistically significant (df = 12, AIC = 240.28, Estimate = −758.44, *t* = −2.16, *p* = .05). Females produced statistically significantly more seeds also per flower (3.51 ± 0.47 seeds per flower) than the hermaphrodites (1.81 ± 0.40 seeds per flower) (df = 12, AIC = 23.12, Estimate = −1.66, *t* = −0.10, *p* < .0001). Sex affected the ratio of wilted flowers to schizocarps (distinguished from flowers by missing seeds or swollen ovaries and elongated stigma), between females (6.8% of all flowers did not develop into schizocarps) and hermaphrodites (26% of all flowers did not develop) (df = 12, Estimate = 1.45, *t* = 2.76, *p* = .02).

Bumblebee visitation rate per flower per hour was positively related to the mean number of seeds produced per flower in a plant (df = 10, AIC = 18.9, Estimate = 0.02, *t* = 2.29, *p* = .05). Visitation rate by Syrphidae was also positively related to the mean production of seeds per flower (df = 10, AIC = 19.03, Estimate = 0.03, *t* = 0.01, *p* = .05), but *Apis* visitation rate did not have any statistically significant relationship (df = 10, AIC = 24.123, Estimate = 0.01, *t* = 0.70, *p* = .50). The bumblebee visitation rate reduced the ratio of undeveloped flowers to developed schizocarps marginally significantly (df = 10, Estimate = −0.04, *t* = −2.34, *p* = .04). Also, Syrphidae visitation ratio had a statistically significant negative effect on the ratio of undeveloped flowers to developed schizocarps (df = 10, Estimate = −0.05, *t* = −3.0, *p* = .01), but *Apis* had no effect on the undeveloped‐to‐developed schizocarps ratio (df = 10, Estimate = −0.03, *t* = −1.4, *p* = .18).

## 4 DISCUSSION

Although sexual dimorphism has arisen in several distinct genera (Miller & Venable, 2003; Thomson & Brunet, 1990), the underlying evolutionary effectors are not clear (Charlesworth, 1981; Delph et al., 1996; Thomson & Brunet, 1990). To explain dimorphism between the sexes, arguments for non‐adaptive (reviewed by Delph et al., 1996), anti‐selfing (Baker, 1959; Kawagoe & Suzuki, 2003; Wilmer, 2011), and resource allocation hypotheses (Ashman, 1994; Chang, 2006; Delph et al., 1996; Eckhart, 1992) have been forwarded. The morphology of a flower is a compromise between different selection pressures (Galen, 1999). Larger flowers often receive higher visitation rates and have been proposed to evolve due to directional selection promoting increased floral attraction (Martin, 2004; Stanton & Preston, 1988). Visitation rates have been frequently shown to be positively linked with flower size (Bond & Maze, 1999; Martin, 2004; Van Etten & Chang, 2014). However, the reverse has not been documented previously: how small, visually unattractive flowers could make up for the loss of visitation rates.

The size of the sex organs in the flower plays a crucial role in the pollination probability. Due to developmental constraints, corolla size in a flower increases in size in symmetry with the other parts of a flower (Moyroyd & Glover, 2017; Paterno et al., 2020). In *G. sylvaticum* flowers, the smaller petal size is associated with smaller style length and larger petals with longer styles (Asikainen & Mutikainen, 2005a). When the stigma is in the receptive phase, the style is typically longer in hermaphrodite flowers than in females (Asikainen & Mutikainen, 2005a). The long style length in hermaphrodite flowers has positive and negative effects on reproduction. The hermaphrodite stigma in the receptive phase protrudes over the anthers. This has positive effects as a means of prevention of autogamy in *Geranium* species (Konarska & Mazierowska, 2020; Philipp, 1985) in addition to the partial protandry in this species (Asikainen & Mutikainen, 2005a; Varga, Nuortila, & Kytöviita, 2013). However, the long style length has negative effects on the female function in hermaphrodites as it reduces the probability of pollen transfer on stigmas by pollinators as is demonstrated in this study. The style length has been shown to have a relatively narrow optima for pollen deposit and pollinator contact probability in *Brassica napus* flowers (Cresswell, 2000).

Larger flowers are advantageous in male function in the way of pollen transport from hermaphrodite flowers (Ashman, 1992). In agreement with our study, Ashman (1992) found that, although longer petals contributed to a better pollen export, the petal length was a poor predictor of pollen deposition. Concluding from the contacts to *G. sylvaticum* reproductive organs in our study, pollen display in hermaphrodites matched stigma display in hermaphrodite flowers poorly. In contrast, female flower morphology was a better match to the hermaphrodite pollen display. Accordingly, different aspects of morphology promote different sexual functions. Hermaphrodite morphology is adapted to pollen export (Ashman, 1992; Asikainen & Mutikainen, 2005a; Bond & Maze, 1999). In females, flower morphology that maximizes pollen receipt on stigma according to hermaphrodite pollen display should be selected because it is the sole function of the female flowers.

The small size of female flowers is often explained by the resource re‐allocation hypothesis stating that the energy and nutrient investment difference between hermaphrodite and female flowers may be allocated to seed production (Ashman, 1994; Chang, 2006; Eckhart, 1992). In this work, we challenge the non‐adaptive and resource re‐allocation hypotheses in explaining the floral dimorphism in gynodioecious plants. We specifically tested the “pollination hypothesis” that flower size variation in *G. sylvaticum* is adaptive because it enhances probability of a visitor contacting stigma, and thus promotes pollination probability in females. We stress that we did not measure pollen deposition, but probability of pollen deposition. We base this estimate on the assumption that only when an insect makes a ventral contact with the receptive stigma lobes, pollen is deposited. In the case when the receptive stigma lobes are not contacted, pollen cannot be transmitted. In support of the pollination hypothesis, the stigmas in the small female flowers were more likely to be contacted by visitors than the stigmas in hermaphrodite plants. This indicates that the balance between visitor attraction and consequent pollen transmission on one hand and pollen deposition on stigmas on the other hand may act as drivers in *G. sylvaticum* sexual dimorphism.

The stepping‐stone hypothesis for the evolution of dioecy requires that the hermaphrodites in a gynodioecious population are biased toward maleness and ultimately lose their female role (Lloyd, 1976; Spigler & Ashman, 2012). One of the explanations for this is the aggravated competition for females through the male function (Lloyd, 1976). Consequently, hermaphrodites in gynodioecious populations are expected to be biased toward maleness (Goldman & Wilson, 1986; Spigler & Ashman, 2012). Assuming a similar number of visits, although smaller than the hermaphrodite flowers, the female flowers are more likely to be pollinated during a single visit than the hermaphrodite ones according to our data. As *G. sylvaticum* female flowers have been shown to receive fewer visits than hermaphrodites previously (Asikainen & Mutikainen, 2005a; Varga & Kytöviita, 2010) and in this work, this estimate is conservative. This is supported also in other studies, as—although hermaphrodite *G. sylvaticum* flowers do in general receive more visits than females (Asikainen & Mutikainen, 2005a; Varga & Kytöviita, 2010)—the fruit set is frequently lower in hermaphrodites than in females (Asikainen & Mutikainen, 2005b; Varga & Kytöviita, 2010, present study). Consequently, our work supports the stepping‐stone hypothesis as hermaphrodite flowers had much lower pollination probability than female flowers during a single visit and lower seed set suggesting lower fitness gains through female function in hermaphrodites.

Flowers are subject to both directional and disruptive selection (Galen, 1999; Galen et al., 1987). Directional selection occurs when, for example, a trait of a plant positively affects pollinator visitation rates creating a selective pressure for increase in the expression of such trait (e.g., see Galen, 1989). In the case of *G. sylvaticum*, sexual dimorphism is the result of disruptive selection fueled by the different flower size optima for female and male fitness. The disruptive selection agents are the pollinating insects that behave differently in *G. sylvaticum* flowers of different sexes. The fact that we observed disruptive selection between the sexes of *G. sylvaticum* also supports the gynodioecy–dioecy pathway hypothesis (Dufay et al., 2014; Spigler & Ashman, 2012). Generally, floral characteristics such as showiness are promoted as the plants benefit from increased number of visitations (Martin, 2004; Van Etten & Chang, 2014), but disruptive selection by insect behavior may help explain the evolution of sexually dimorphic flowers. If the differential selection pressures on the sexes persist, *G. sylvaticum* may evolve toward dioecy.

Although we note that disruptive selection drives floral sexual dissimilarity in size, floral constancy may limit the evolutionarily stable degree of dimorphism. Due to the floral constancy behavior of the insects (Waser, 1986), the flowers need to be perceived as similar enough to be constantly visited. The scarcity of females can also cause minority disadvantage (Levin, 1972), which further reduces the visitation rates in *G. sylvaticum* females (Van Etten & Chang, 2014). We only distinguished the flowers by their size which was sex‐specific and the presence/absence of anthers. In addition to corolla size, several other factors such as odor, color, and their relations to bee memory and handling skills (Chittka et al., 1999; Ishii & Masuda, 2014; Waser, 1986) could be responsible for floral constancy. Bees possess notable olfactory discrimination abilities (Laska et al., 1999). Pollinators relying on cues such as odor or the color spectra could explain why, e.g., the bumblebees do not always discriminate between the sexes of *G. sylvaticum* (Asikainen & Mutikainen, 2005a) despite the dimorphism in size and the fact that females produce less nectar (Varga, Nuortila, & Kytöviita, 2013). In this experiment, bumblebee visitation rates were notably higher in hermaphrodites suggesting that at least occasionally bumblebees may favor the more rewarding sex. The morphological size dimorphism in *G. sylvaticum* may be furthered if the floral constancy of pollinators is more tightly linked to factors other than the size of flowers.

The pollen and stigma displays are linked via the morphology and behavior of the pollinator insects. Insect morphology imposes selective pressure on flowers to match the reproductive displays of the sexes. In a previous study on *Cucurbita maxima*, bumblebees carried considerably more pollen on their bodies than honeybees (Kamo et al., 2022). Due to their pollen transport capacity and behavior within the flower, bumblebees also effectively deposited more pollen than honeybees or other floral visitors (Kamo et al., 2022). Honeybee, *Apis mellifera*, is a non‐native farmed insect in Finland. Our results suggest that although pollinating to some degree, it is inferior to native pollinators. Honeybee visitation rates did not influence the mean seed production per flower in our study plants, but *Bombus* and Syrphidae visitation rates did. Syrphid flies have been shown to be the most common floral visitors in *G. sylvaticum* (Bauman et al., 2021; Varga & Kytöviita, 2010). However, in our study Syrphid flies were likely to pollinate female flowers and even then only to a relatively small degree. *Bombus* had the highest likelihood of all of the visitor groups to contact *G. sylvaticum* anthers and stigma. This work supports our previous work that members of the genus *Bombus* are the primary pollinators of *G. sylvaticum* (Varga & Kytöviita, 2010). Bumblebees were noted to be behaviorally and morphologically effective pollinators also in a closely related hermaphroditic *Geranium* species (Kandori, 2002). In contrast to hermaphrodites, the small size of female stigma facilitated stigma contacts to a small degree also by honeybees and Syrphid flies. Because hermaphrodites were only pollinated by *Bombus*, but females by several insect groups, sexual size dimorphism could assure reproduction in both sexes in the face of fluctuating pollinator populations.

Although effective pollinators, bumblebees were the only visitor group that had the potential to effectuate self‐pollination in the hermaphroditic flowers (in 5.4% of all visits by *Bombus* the insect touched an anther and then stigma in the same flower in a manner that could cause pollination). Avoidance of inbreeding is one of the mechanisms that has been proposed to drive gynodioecy (Baker, 1959; Charlesworth & Charlesworth, 1987). The effects of inbreeding vary between species and populations (Keller & Waller, 2002). Some inbreeding depression in *G. sylvaticum* has been observed in terms of lower germination rate in self‐pollinated vs. crossed offspring (Varga, Vega‐Frutis, & Kytöviita, 2013). Given that on average bumblebees visit 2–3 flowers within a plant (Asikainen & Mutikainen, 2005a), and that there is a small degree of anther‐to‐stigma contacts in the same flower (this study), the self‐pollination rate effectuated by *Bombus* may have some consequences. It should be noted, however, that the effects of inbreeding would be diminished in gynodioecious populations where higher proportion of offspring would be the result of crossbreeding (Baker, 1959). Altogether, it is likely that both the pollination hypothesis and the anti‐selfing hypothesis (Baker, 1959; Kawagoe & Suzuki, 2003) explain the dimorphism in gynodioecious populations.

## 5 CONCLUSIONS

Our results demonstrate disruptive selection in flower size and are in line with the stepping‐stone hypothesis in explaining gynodioecy in *G. sylvaticum*. Supporting our “pollination hypothesis” dimorphism in *G. sylvaticum* seems to be adaptive in terms of optimizing female and male fitness in females and hermaphrodites, respectively. The two sex morphs are linked and selected by pollinator behavior.

Various insect species visited the flowers, but it is apparent that the two sex morphs are most efficiently pollinated by bumblebees whose visitation rates were also linked with seed production. The female function of hermaphrodites was dependent on bumblebees, whereas the female function in females was supported by several insect groups. This may promote sexual dimorphism depending on local insect fauna and its fluctuations and should be studied further. Farmed honeybee*s* provided inferior pollination services compared to native pollinators and did not link with seed production or the ratio of wilted flowers to developed schizocarps. Altogether these results highlight the importance of pollinator diversity and of bumblebees in particular in plant sexual reproduction.

## AUTHOR CONTRIBUTIONS

**Jaakko O. S. Soininen:** Conceptualization (equal); data curation (lead); formal analysis (lead); funding acquisition (lead); investigation (equal); methodology (equal); project administration (supporting); resources (supporting); software (lead); validation (equal); visualization (lead); writing – original draft (equal); writing – review and editing (equal). **Minna‐Maarit Kytöviita:** Conceptualization (equal); data curation (supporting); formal analysis (supporting); funding acquisition (supporting); investigation (equal); methodology (equal); project administration (equal); resources (lead); software (supporting); supervision (lead); validation (equal); visualization (supporting); writing – original draft (equal); writing – review and editing (equal).

## Data Availability

Data are deposited in the JYX repository (Library of the University of Jyväskylä). https://doi.org/10.17011/jyx/dataset/84567.
